# US Older Adults That Consume Avocado or Guacamole Have Better Cognition Than Non-consumers: National Health and Nutrition Examination Survey 2011–2014

**DOI:** 10.3389/fnut.2021.746453

**Published:** 2021-10-14

**Authors:** Feon W. Cheng, Nikki A. Ford, Matthew K. Taylor

**Affiliations:** ^1^Avocado Nutrition Center, Mission Viejo, CA, United States; ^2^Medical Center Department of Dietetics and Nutrition, University of Kansas, Kansas City, KS, United States; ^3^Alzheimer's Disease Center, University of Kansas, Fairway, KS, United States

**Keywords:** cognitive performance, older adults, avocado, brain health, NHANES

## Abstract

**Purpose:** The goal of this study is to examine how avocado relates to cognitive function among older adults.

**Methods:** A total of 2,886 National Health and Nutrition Examination Survey 2011–2014 participants aged 60 or older met the eligibility criteria and were included of our cross-sectional study. Participants were binarily classified as avocado consumers (i.e., reported consuming any avocado/guacamole in either 24-h dietary recalls) or non-consumers. Cognitive performance was evaluated with: Consortium to Establish a Registry for Alzheimer's disease (CERAD)—immediate and delayed recall (IWR/DWR), the Animal Fluency test, and the Digit Symbol Substitution Test. We calculated the education-dependent z-scores for each subject because education level can impact cognitive function. Global cognitive score, an average of the z-scores for each cognitive test, was calculated in participants who had completed all four tests. To account for relevant covariates, we tested for mean differences in cognition between consumers and non-consumers using independent sample *t*-tests and ANCOVA, special cases of ordinary least squares regression.

**Results:** Avocado consumers had significantly better cognitive scores across all cognitive tests and the global cognition score (*p* < 0.05) in the unadjusted model. Some mean differences attenuated after adjusting for potential confounders, but others remained significant. Compared to non-consumers, avocado consumers had significantly higher z-scores of 0.15, 0.15, and 0.11 for CERAD IWR and DWR, and global cognition score, respectively (all *p* < 0.05 in adjusted models).

**Conclusion:** Avocado consumption was associated with significantly better IWR, DWR, and the overall global cognition score, which remained significant when controlling for all relevant confounders.

## Introduction

Brain health is becoming an increasingly important research topic, especially in the older adult population, as it is estimated that diagnosis of Alzheimer's Disease (AD) will triple by 2060 from 2017 (1–3). Many of the known modifiable risk factors for cognitive decline and dementia are cardiovascular-related (1, 4). Some of those risk factors include high cholesterol, hypertension, obesity, diabetes, and tobacco use. Improved nutrition can help alleviate many of these modifiable risk factors and may directly impact the brain (1, 4).

There have been studies showing the potential benefits of single food items and dietary patterns on brain health. For instance, greater adherence to the Mediterranean diet has been associated with slower cognitive decline and cognitive impairment (5). In a cross-sectional analysis of the National Health and Nutrition Examination Survey (NHANES) survey (2011–2014), Taylor et al. found that greater Mediterranean Diet adherence was associated with better cognitive performance among individuals aged 60 years and older (6). Similarly, the German Longitudinal Cognitive Impairment and Dementia Study found that those with greater adherence to the Mediterranean Diet had better memory in older adults (7). Although avocados are not part of a traditional Mediterranean diet, consumption is dramatically increasing worldwide, and their nutrient profile aligns strikingly well with the Mediterranean dietary pattern.

A medium avocado fruit provides a lipid profile almost identical to olive oil plus 16% daily value (DV) niacin and riboflavin, 24% DV vitamin B6, 32% DV folate, 44% pantothenic acid, 408 ug of the carotenoid lutein, 36% of the DV for fiber, 16% DV vitamin C, and 20% DV vitamin E (8, 9). Single food components with similar fatty acid profiles to avocados, such as extra virgin olive oil and nuts, have been shown to correlate with cognition (10–12). Yet, few studies have investigated the association between avocados and cognition (13, 14). The two existing studies are limited in sample size and only one of them examined older adults, which may not be representative of the general older adult population. Both studies provided one whole avocado a day for 12 weeks or 6 months, which does not reflect habitual intake of avocado within the US population. Therefore, it's important to understand the role of this understudied but popularly consumed food on cognitive function using a large nationally representative health and nutrition survey. Specifically, this study aims to examine how avocado relates to cognitive function among individuals aged 60 or older from the Natonal Health and Nutrition Examination Survey (NHANES) 2011–2014.

## Materials and Methods

### Study Population

This cross-sectional study was conducted using NHANES, a publicly available dataset. NHANES is a program within the National Center for Health Statistics, a part of the Centers for Disease Control and Prevention^1^. It used a multistage, probability sampling design to select participants representing the non-institutionalized US population (15). It also oversampled some subgroups to allow for a better estimate for those subgroups. All NHANES participants were provided informed consent, and the National Center of Health Statistics Research Ethics Review Board reviewed and approved NHANES protocols.

**Figure 1:**
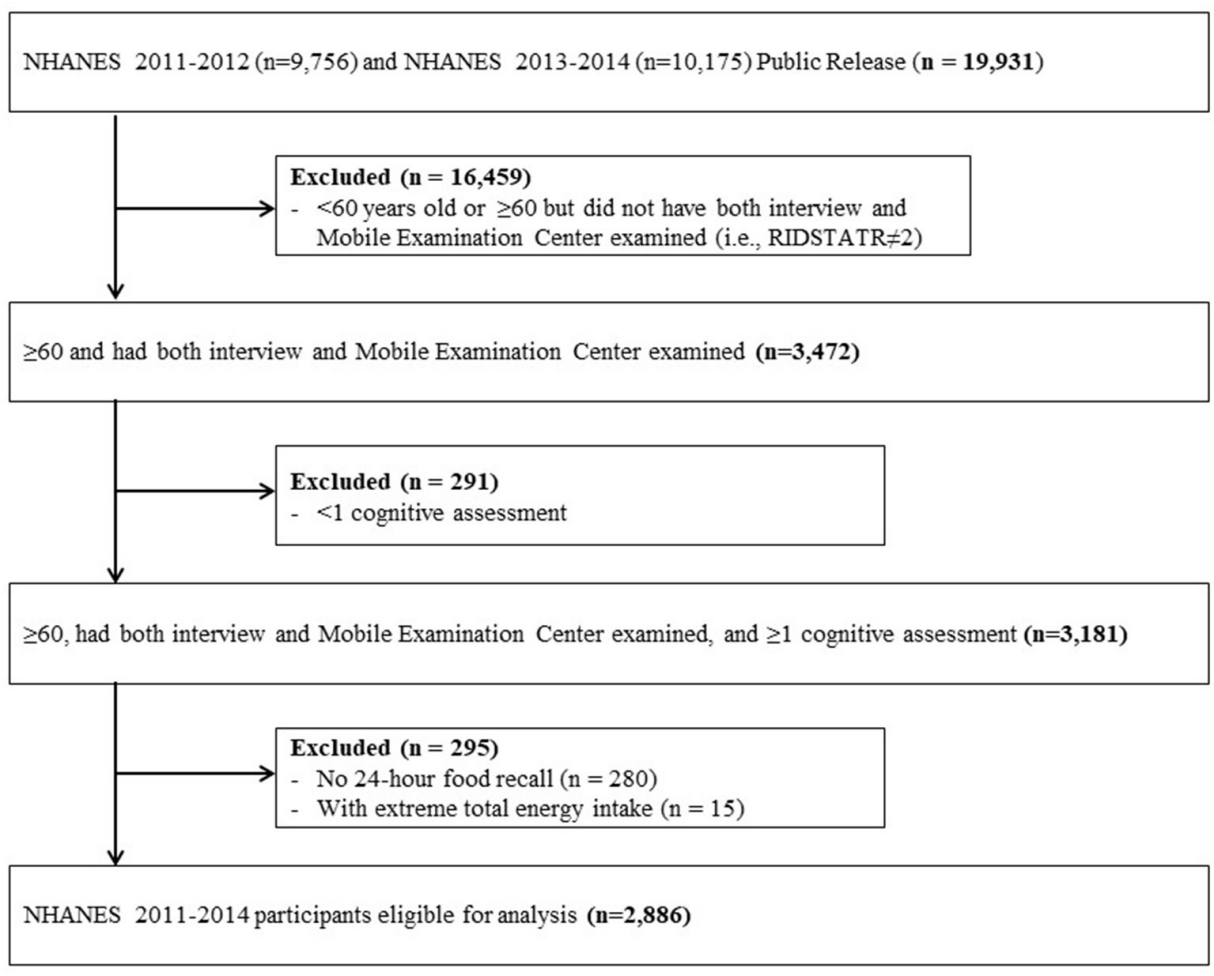
Flow chart of eligible National Health and Nutrition Examination Survey (NHANES) participants included in this analysis.

Cognitive tests were administered only in the 2011–2012 and 2013–2014 cycles to adults 60 years or older; thus we restricted our analysis to participants in this age group and survey waves (*n* = 3,472) (16). Figure 1 displays the flow chart of eligible NHANES participants for this study. Subjects were included if they had at least one cognitive assessment and one 24-h food recall. Those with extreme total energy intake (e.g., women: <500 or >5000 kcal/day for women and <500 or >8,000 kcal/day for men) were further excluded (17). A total of 2,886 NHANES 2011–2014 participants were appropriate for this study.

### Dietary Assessment

Trained interviewers collected 24-h dietary recalls. The first recall was conducted in the Mobile Examination Center (MEC), and the second recall was conducted over the phone 3–10 days later. More details of the interview process are described elsewhere (18).

Food codes from the USDA Food and Nutrient Database for Dietary Studies were used to identify avocado (63105010) and guacamole (63409010) consumers. Participants were binarily classified as consumers or non-consumers. Avocado consumers were defined as participants who reported consuming any avocado/guacamole in either 24-h dietary recalls. Avocado non-consumers were defined as participants who didn't report eating any avocado/guacamole during either 24-h dietary recalls.

### Cognitive Function

NHANES participants aged 60 years or older not requiring a proxy informant in the 2011–2014 cohorts completed cognitive tests at home and in the MEC. The assessments were conducted in the following order: Consortium to Establish a Registry for Alzheimer's disease (CERAD)—immediate recall (IWR), the Animal Fluency test (AFT), the Digit Symbol Substitution Test (DSST), and CERAD—delayed recall (DWR). Cognitive tests were available in different languages, including English, Spanish, Korean, Chinese, and Vietnamese.

The CERAD assessment evaluates IWR and DWR of new verbal learning (19). Participants completed three learning trials where the trained interviewer showed words to the participants on flip cards. During each learning trial, the participants read the words out loud, one at a time. After flipping through all 10 cards, the participant had 90 s to remember as many words as possible. The CERAD score ranged from 0 to 10 for each learning trial; each correctly recalled word earned one point. The CERAD IWR score was an average of correctly recalled words from the three trials. After the AFT and DSST assessments, one delayed recall was conducted, about 8–10 min after the initial word cards were presented.

The AFT test is a test of executive function, assessing verbal fluency by asking subjects to audibly list as many animals as possible within 1 min (20). As a practice trial, participants were asked to list aloud three clothing items. If they could not accomplish the test task, they did not continue with the AFT assessment. The calculated score equals the number of correct animals named.

The DSST is a subtest of the Wechsler Adult Intelligence Scale, Third Edition, which tests processing speed and attention using a paper form (21). The top of the page shows numbers one through nine with unique symbols associated with each number. Subjects were given 2 min to draw corresponding symbols in the 133 numbered boxes. The score ranged from 0 to 133; each point represents a correct match. Participants did a practice test, and those who were unable to complete it without the interviewer's help did not continue the DSST.

Because education level has been shown to impact cognitive function significantly, we calculated the education-dependent z-scores for each subject (22). Participants were first grouped by their educational levels (i.e., <9th grade, 9–11th grade, high school grad/GED or equivalent, some college or AA degree, and college graduate or above). We then calculated participants' education-dependent z-scores for each cognitive test with the mean and standard deviation derived from each education level group. Finally, a global cognitive score, taken as the average of the z-scores for each cognitive test, was calculated in participants who had completed all four tests.

### Covariates

The following variables were assessed for confounding: age (continuous), gender (male and female), the ratio of family income to poverty (continuous), race (Mexican American, other Hispanic, non-Hispanic White, non-Hispanic Black, non-Hispanic Asian, and other race—including multi-racial), marital status (married, widowed, divorced, separated, never married, and living with a partner), smoked at least 100 cigarettes in life (yes and no), had at least 12 alcohol drinks per 1 year (yes and no), work activity (vigorous, moderate, and other), recreational activities (vigorous, moderate, and other), body mass index (continuous), Mediterranean Diet score (continuous), self-reported physician diagnosis of prediabetes or diabetes (yes and no), self-reported physician diagnosis of coronary heart disease (yes and no), self-reported physician diagnosis of high blood pressure (yes and no), and self-reported physician diagnosis of stroke (yes and no).

The work and recreational activities variables were calculated using the Global Physical Activity Questionnaire, which asks two sets of similar questions to characterize typical work activity and typical recreational activity. The first set asks whether the participant regularly engages in 10 min of continuous vigorous activity and the other set determines whether the participant regularly engages in 10 min of continuous moderate activity. Subjects were classified as engaging in “vigorous work activity” if they answered “yes” to the vigorous work activity question, “moderate work activity” if they answered “no” to vigorous work activity and “yes” to moderate work activity, and “other” if they answered “no” for both questions. The same approach was used for questions relating to recreational activity.

Calculation of the Mediterranean Diet score in this sample has been previously described (6) using a modified version of Sofi, et al.'s 18-point Mediterranean Diet index (Sofi MedD Score) (23). Briefly, the Sofi MedD Score consists of 9 groups: fruit, vegetables, legumes, cereals, fish, meat, dairy, alcohol, and olive oils. Each group was assigned either 0, 1, or 2, depending on an individual's intake. Except for meat, dairy, and alcohol, a greater score represents a higher consumption of each food group.

### Statistical Analysis

For all statistical analyses, we used sampling weights designed to account for multiple cycles of complex, multistage surveys (15). We performed descriptive statistics to show the baseline characteristics. Avocado consumers and non-consumers characteristics were compared using independent sample *t*-tests and chi-square tests. We compared means for education-dependent cognitive performance between the two avocado consumption groups using unadjusted and covariate-adjusted ordinary least squares (OLS) regression models (i.e., independent sample *t*-test and ANCOVA). Model 1 was unadjusted. Model 2 was adjusted for age, gender, the ratio of family income to poverty, race, and marital status. Model 3 further adjusted for smoking status, alcohol consumption, work activity, recreational activities, BMI, Mediterranean Diet score, self-reported physician diagnosis of prediabetes or diabetes, self-reported physician diagnosis of coronary heart disease, self-reported physician diagnosis of high blood pressure, and self-reported physician diagnosis of stroke. Covariates were selected based on previous research showing potential association between these covariates and exposure and/or outcomes (6, 16, 17, 24–27). There were no indications of multicollinearity in adjusted regression models (i.e., variance inflation factor ≤2.5 and tolerance >0.2), and model assumptions were tested using residual analyses (e.g., residual histograms and quantile-quantile plots). All analyses were performed with SAS 9.4 (SAS Institute Inc., Cary, NC), and the level of significance was considered at *p* < 0.05.

### Protocol Registration and Checklist

We developed and registered our study protocol at: osf.io/yjbdn before data analysis (28). In addition, this study also followed the Strengthening the Reporting of Observational Studies in Epidemiology (STROBE) checklist to ensure the quality of the research.

## Results

Of 19,931 NHANES 2011–2014 participants, 2,886 individuals met the criteria for this study (Figure 1). Approximately 7% of them were avocado consumers with an average (standard error) avocado and guacamole consumption of 73.61 (7.25) and 53.37 (9.41) grams, respectively. In general, avocado consumers were younger, more likely to be married, had a higher family income to poverty ratio, and educational level (Table 1). They also had a lower BMI, engaged in more vigorous or moderate recreational activities, and had a higher diet quality, as measured by the Mediterranean Diet Score (Table 1).

**Table 1:** Baseline characteristics of NHANES participants by avocado/guacamole consumer vs. non-consumer (*n* = 2,886)^*^.

**Avocado/guacamole consumer^†^**	**All**	**No**	**Yes**	***P***
	**(*n =* 2,886)**	**(*n =* 2,693)**	**(*n =* 193)**	
Age (yr)	69.3 (0.2)	69.4 (0.2)	67.9 (0.5)	0.03
Female, %	53.8	53.2	62.0	0.08
Family income to poverty ratio	3.1 (0.1)	3.1 (0.1)	3.7 (0.2)	0.003
Race/ethnicity, %				0.002
Mexican American	3.4	3.3	4.6	
Other hispanic	3.7	3.4	7.9	
Non-hispanic white	79.5	79.4	80.1	
Non-hispanic black	8.5	9.0	2.8	
Non-hispanic Asian	3.0	3.0	3.4	
Other race-including multi-racial	1.9	1.9	1.2	
Marital status, %				<0.001
Married	62.6	61.5	76.0	
Widowed	16.9	17.8	5.4	
Divorced	12.3	12.4	11.1	
Separated	1.2	1.3	0.4	
Never married	4.3	4.1	6.8	
Living with partner	2.6	2.8	0.3	
Education, %				<0.001
<9th grade	6.0	6.2	4.4	
9–11th grade	10.3	10.7	5.5	
High school grad/GED or equivalent	22.2	22.8	14.4	
Some college or AA degree	31.5	31.8	27.4	
College graduate or above	30.0	28.5	48.2	
BMI (kg/m^2^)	29.1 (0.2)	29.3 (0.2)	27.5 (0.7)	0.02
Smoker, %^‡^	50.4	50.6	47.2	0.59
Alcohol drinker, %^§^	72.5	71.6	83.7	0.005
Work activity, %				0.99
Vigorous	12.9	12.8	13.4	
Moderate	21.9	21.9	22.1	
Other	65.2	65.2	64.5	
Recreation activities, %				0.0005
Vigorous	11.4	10.5	22.5	
Moderate	33.8	33.4	38.0	
Other	54.8	56.0	39.5	
Prediabetes or diabetes, %^¶^	30.2	30.6	25.7	0.34
Coronary heart disease, %^¶^	9.7	9.9	6.3	0.17
Stroke, %^¶^	6.6	6.8	4.5	0.47
Hypertension, %^¶^	59.4	59.7	55.7	0.42
Energy intake, kcals/day	1,870.7 (18.2)	1,862.5 (19)	1,973.0 (65)	0.12
Mediterranean Diet Score	5.3 (0.1)	5.2 (0.1)	6.2 (0.2)	0.0003
Raw cognitive scores				
CERAD immediate learning	6.5 (0.1)	6.5 (0.1)	7.1 (0.1)	<0.001
CERAD delayed recall	6.2 (0.1)	6.1 (0.1)	7.0 (0.1)	<0.001
Animal fluency test	18.1 (0.2)	17.9 (0.2)	20.1 (0.6)	0.0008
Digit symbol substitution test	52.4 (0.5)	51.8 (0.6)	58.9 (1.7)	0.0005

*BMI, body mass index; CERAD, Consortium to Establish a Registry for Alzheimer's disease*.

* *Means (standard errors) for continuous variables and percentages for categorical variables*.

† *Avocado/guacamole consumers were identified as NHANES 2011–2014 participants who reported consuming any amount of avocado or guacamole during the 24-h dietary recall*.

‡ *Smoker is defined as those who had at least 100 cigarettes in life*.

§ *Alcohol drinker is defined as those who had at least 12 alcohol drinks/1 year*.

¶ *Self-reported physician diagnosis*.

**Figure 2:**
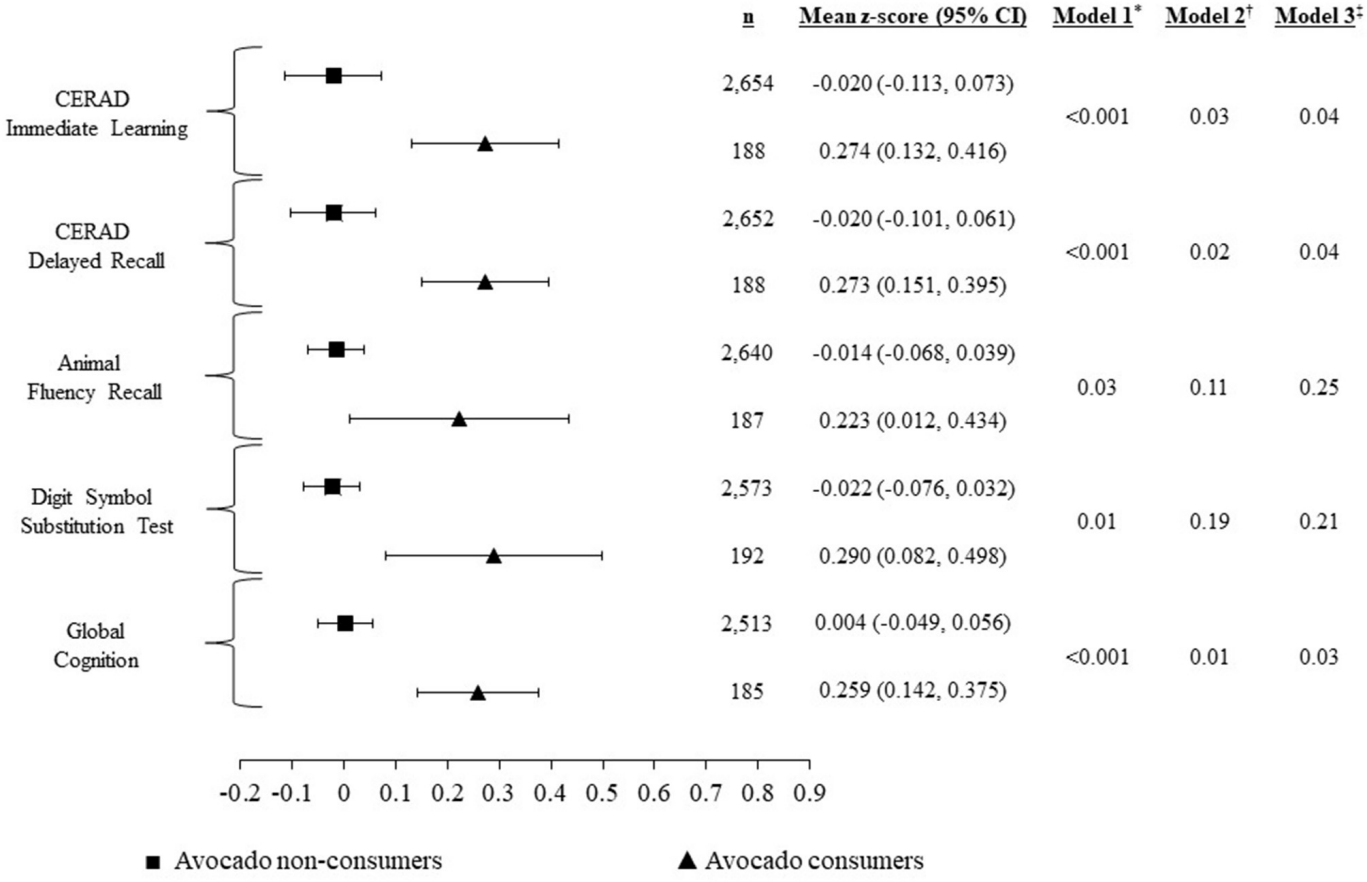
Mean z-scores, corresponding cognitive test 95% confidence intervals, and p-values for each cognition test. CERAD, Consortium to Establish a Registry for Alzheimer's disease. *Model 1 is unadjusted. Sample sizes for each cognitive test were: Consortium to Establish a Registry for Alzheimer's Disease–Immediate Learning (*n* = 2,842); Consortium to Establish a Registry for Alzheimer's Disease-Delayed Recall (*n* = 2,840); Animal Fluency Test (*n* = 2,827); Digit Symbol Substitution Test (*n* = 2,765); Global cognition (*n* = 2,698). ^†^Model 2 adjusted for age, gender, ratio of family income to poverty, race, and marital status. Sample sizes for each cognitive test were lower because of missing covariates: Consortium to Establish a Registry for Alzheimer's Disease-Immediate Learning (*n* = 2,621); Consortium to Establish a Registry for Alzheimer's Disease–Delayed Recall (*n* = 2,619); Animal Fluency Test (*n* = 2,608); Digit symbol substitution test (*n* = 2,553); Global cognition (*n* = 2,491). ^‡^Model 3 further included adjusted for smoking status, alcohol consumption, work activity, recreational activities, BMI, Mediterranean Diet score, self-reported physician diagnosis of prediabetes or diabetes, self-reported physician diagnosis of coronary heart disease, self-reported physician diagnosis of high blood pressure, and self-reported physician diagnosis of stroke. Sample sizes for each cognitive test were lower because of missing covariates: Consortium to Establish a Registry for Alzheimer's Disease-Immediate Learning (*n* = 2,532); Consortium to Establish a Registry for Alzheimer's Disease–Delayed Recall (*n* = 2,531); Animal Fluency Test (*n* = 2,521); Digit Symbol Substitution Test (*n* = 2,477); Global Cognition (*n* = 2,417).

Figure 2 illustrates the mean education-dependent z-scores, corresponding cognitive test 95% confidence intervals, and *p*-values for avocado consumers and non-consumers. In the unadjusted model, avocado consumers had significantly better cognitive performance across all cognitive tests and the global cognition score (*p* < 0.05) (Figure 2 and Supplementary Table 1). Some mean differences attenuated after adjusting for potential confounders (models 2 and 3), but others remained significant. In the fully adjusted model 3, compared to non-consumers, avocado consumers had significantly higher *z*-scores of 0.15, 0.15, and 0.11 for CERAD IWR and DWR, and global cognition scores, respectively (all *p* < 0.05).

Supplementary Table 1 presents complete regression statistics for all three of our OLS models using avocado consumer vs. non-consumer as the primary independent variable.

## Discussion

This study was the first to compare the differences in cognitive function between avocado consumers and non-consumers in an observational dataset of older Americans. Avocado consumers had significantly better scores for the CERAD IWR and DWR tests and the overall global cognition score and remained significant when controlling for relevant confounders (in all adjusted models). Our study found that avocados may have an impact independent of a number of covariates, including diet quality (per model 3), which is associated with slower cognitive decline and cognitive impairment (5). While the AFT and DSST scores were significantly better for avocado consumers in our unadjusted model, the significance was attenuated in the adjusted models. Given avocado consumption is growing dramatically worldwide, and research has shown the cardiometabolic benefits of eating avocados through the effects of various dietary components, phytochemical, and nutrients that avocados include as discussed by Silva Caldas et al. (29–34), it's relevant to study the cognitive benefits of avocado intake within a large population cohort.

We found that consuming avocado was most strongly related to better memory performance. Among all participants, avocado consumers recalled an average of 1.8 more words across the three learning trials for the IWR and 0.9 more words on the DWR. For cognitively normal individuals of this age group, the average performance by avocado consumers on these tasks was at the median for suggested normative performance while average performance by non-consumers was at the bottom of the suggested interquartile range (35). This is critical because memory is the most common complaint in older adults and likely the first cognitive domain to be affected in age-related neurodegenerative disease (i.e., AD and Mild cognitive impairment) (36, 37). Two previous studies investigated the effects of avocado on cognitive function and found some positive benefits, similar to our study. Scott et al. randomized healthy older adults into a 6-month trial where subjects consumed either one avocado or one cup of potato or chickpeas per day (13). The control foods were selected to limit the intake of lutein, a nutrient hypothesized to contribute to cognitive performance and present in the fresh avocado pulp. A battery of cognitive tests evaluated different cognitive domains, including attention (Choice Reaction Time and Rapid Visual Information Processing), visual memory (Delayed Match to Sample, Paired Associates Learning), and executive function, working memory, and planning tests (Spatial Span, Spatial Span Reverse, Spatial Working Memory, and Stockings of Cambridge). The avocado group had improvements in working memory. Additionally, as lutein accumulated in the eye's macula, working memory and efficiency of approaching a problem also improved. Similarly, we found that those consuming avocado scored higher on the CERAD IWR and DWR memory tests. Evidence suggests that avocados could benefit cognition across the lifespan, potentially influencing different cognitive domains at different life stages. The other randomized controlled trial conducted by Edwards et al. found that 84 overweight and obese adults performed better on a cognitive test of attentional inhibition when consuming fresh avocado daily for 12 weeks than an isocalorically controlled meal (14). To evaluate cognitive function, they used the Flanker task, oddball task, and Nogo task. They found that the avocado group improved accuracy in the Flanker task, even when accounting for numerous confounders. The other cognitive measures were not significantly changed. Although the tests used in this study differ from our current study, it is consistent that avocado consumption seems to benefit specific domains in cognitive function vs. all the domains. Our study suggests that additional clinical trial in older adults is necessary to elucidate effects of an avocado intervention.

There are several potential mechanisms to support the observed associations between avocado intake and cognitive function. One Hass avocado contains ~369 micrograms of the carotenoid, lutein (8). Lutein preferentially accumulates in neural tissues and therefore is hypothesized to be involved in neurocognitive function across the lifespan, and numerous studies report lutein-related cognitive benefits (38). Kesse-Guyot et al. observed that a carotenoid-rich dietary pattern was associated with a better overall cognitive score after a 13-year follow-up (39). Two prospective studies reported reduced cognitive aging for individuals consuming higher amounts of lutein and zeaxanthin-containing vegetables (40–42). The relationship between brain lutein concentrations of decedents >98 y at death and premortem measures of cognitive function were assessed in a study by Johnson et al. Among the carotenoids, brain lutein content was reliably related to cognitive test scores (43). Although findings from the RCT (14) did not find a correlation between serum lutein levels and attentional inhibition, it is important to note that macular pigment measures of lutein were not significantly changed. Because it is assumed that lutein deposited in the macula correlates with brain lutein levels, the intervention likely did not signiticantly boost lutein levels in the brain and thus is not responsible for the positive cognitive findings in the study. As a whole food, it is inherently difficult to identify individual avocado nutrients or bioactive compounds that are responsible for the biological benefits observed. Instead, it's plausible that nutrients work additively or synergistically to benefit cognitive measures.

Avocados also contain 13.3 grams of monounsaturated fat and provide vitamins and minerals known to support brain health and cognitive function (5, 8). In addition, the anti-inflammatory and antioxidant effects of monounsaturated fat and its derivatives can help decrease chronic inflammation and oxidative stress, which have been observed in individuals with mild cognitive impairment and AD (44, 45). For example, in a prospective cohort study of women aged 60 years and older, Naqvi et al. found that monounsaturated fat intake was associated with a slower decline in cognitive function, as indicated by the overall cognitive function score (46). Interestingly, monosaturated fat intake was particularly beneficial to the memory and visual domains, similar to what we observed in our analysis.

Avocados contain B vitamins, which have been studied for their potential role in brain health because of their role in homocysteine metabolism (5, 8). Elevated homocysteine level is a risk factor for AD and dementia. B vitamins can help to lower homocysteine levels (47). For example, folate is needed for the remethylation of homocysteine to methionine, and vitamin B_6_ is a cofactor for the enzyme which breaks down homocysteine to sulfate (48). Although results from B vitamin supplementation trials are mixed, observational studies that examined B-vitamins intake and cognitive function have shown some significant benefits (5, 49). For instance, several studies reported that a higher intake of B- vitamins (e.g., folate) was associated with less cognitive decline or less risk for AD and dementia (50–52).

As evidence linking cardiometabolic health with brain health and function continues to grow (53, 54), we also hypothesize that the relationship between avocado intake and better cardiometabolic health may provide explanation for our findings (29–34). For instance, avocados have micronutrients that may benefit blood pressure and avocado interventions have been shown to lower LDL and raise HDL in patients with hypercholesterolemia (30–32, 34) and improve peripheral blood flow acutely after consumption (29, 33).

Our study includes several strengths. First, we used a large nationally representative health and nutrition survey. Second, although we found that avocado consumers tended to be younger, more likely to be married, had a higher educational level and family income to poverty ratio, we adjusted for numerous characteristics in our final model to best understand the specific role of avocado on cognitive measures. One of those key characteristics was education level which is independently correlated with cognitive performance. Thus, we calculated education-dependent z-scores for each cognitive outcome.

Limitations of interpreting these findings include the study's cross-sectional nature, potential residual confounding of observational studies, and the limited number of dietary recalls used to assess avocado intake. For example, one or two 24-h dietary recall(s) may not accurately reflect usual intake. Thus, it would be important to replicate this study using a food frequency questionnaire. Also, since only 7% of the population studied were avocado consumers, we could not complete complementary sensitivity analyses in those with subjective memory complaints. However, avocado intake has dramatically increased from an annual 5 pounds per capita in 2011 to close to 9 pounds per capita in 2020, so the data analyzed in these NHANES cycles may underestimate current avocado consumption trends among older adults, and newer releases of NHANES data may capture a larger sample of avocado consumers.

Although our findings cannot be considered causal, and more studies are needed to confirm these findings, the data suggests a role for avocados in the cognitive function of older Americans. In addition, future studies are warranted to replicate these associations in other populations and examine the mechanism of action.

## Data Availability Statement

Publicly available datasets were analyzed in this study. This data can be found here: The data that support the findings of this study are openly available in the National Health and Nutrition Examination Survey database from the Centers for Disease Control and Prevention at https://wwwn.cdc.gov/nchs/nhanes/Default.aspx.

## Ethics Statement

The studies involving human participants were reviewed and approved by the Research Ethics Review Board at the National Center for Health Statistics. All NHANES participants provided their written informed consent to participate in this study. The patients/participants provided their written informed consent to participate in this study.

## Author Contributions

All authors conception and design, analysis and interpretation of the data, drafting of the article, critical revision of the article for important intellectual content, and also have seen and agreed with the manuscript's contents and met the ICMJE requirements for authorship.

## Funding

This research was supported by the Hass Avocado Board and the National Institutes of Health (K01AG065487).

## Conflict of Interest

NAF and FWC were employed by Hass Avocado Board, Mission Viejo, United States. The remaining author declares that the research was conducted in the absence of any commercial or financial relationships that could be construed as a potential conflict of interest.

## Publisher's Note

All claims expressed in this article are solely those of the authors and do not necessarily represent those of their affiliated organizations, or those of the publisher, the editors and the reviewers. Any product that may be evaluated in this article, or claim that may be made by its manufacturer, is not guaranteed or endorsed by the publisher.

## Footnotes

^1^Centers for Disease Control and Prevention. *National Health and Nutrition Examination Survey*. Survey Methods and Analytic Guidelines. Available online at: https://www.cdc.gov/nchs/nhanes/index.htm (accessed April 25 2021).
