# Supplementary material for: US Older Adults That Consume Avocado or Guacamole Have Better Cognition Than Non-consumers: National Health and Nutrition Examination Survey 2011–2014
Source: Front Nutr. 2021 Oct 14;8:746453. doi: 10.3389/fnut.2021.746453 (PMC8551489; doi:10.3389/fnut.2021.746453)
Supplement: Supplementary file 1 [file Table_1.pdf]

Supplementary table 1. Survey weight-adjusted ordinary least squares regression models assessing the relationship between avocado and guacamole consumer and education-dependent, standardized cognition scores

	Model 1[a]			Model 2[b]			Model 3[c]		
	β	95% CI	*p*	β	95% CI	*p*	β	95% CI	*p*
CERAD Immediate Learning	0.30	0.16, 0.43	<0.001	0.15	0.01, 0.29	0.03	0.15	0.00, 0.29	0.04
CERAD Delayed Recall	0.29	0.15, 0.44	<0.001	0.17	0.03, 0.32	0.02	0.15	0.01, 0.29	0.04
Animal Fluency Test	0.24	0.03, 0.45	0.03	0.14	-0.04, 0.32	0.11	0.11	-0.08, 0.30	0.25
Digit Symbol Substitution Test	0.31	0.10, 0.53	0.01	0.13	-0.07, 0.33	0.19	0.12	-0.07, 0.30	0.21
Global Cognition	0.26	0.14, 0.37	<0.001	0.12	0.03, 0.21	0.01	0.11	0.01, 0.20	0.03

Abbreviations: CERAD, Consortium to Establish a Registry for Alzheimer's disease.

[a]Model 1 is unadjusted. Sample sizes for each cognitive test were: Consortium to Establish a Registry for Alzheimer's Disease–Immediate Learning (n = 2,842); Consortium to Establish a Registry for Alzheimer's Disease–Delayed Recall (n = 2,840); Animal Fluency Test (n = 2,827); Digit Symbol Substitution Test (n = 2,765); Global Cognition (n = 2,698).

[b]Model 2 adjusted for age, gender, ratio of family income to poverty, race, and marital status. Sample sizes for each cognitive test were lower because of missing covariates: Consortium to Establish a Registry for Alzheimer's Disease–Immediate Learning (n = 2,621); Consortium to Establish a Registry for Alzheimer's Disease–Delayed Recall (n = 2,619); Animal Fluency Test (n =2,608); Digit Symbol Substitution Test (n = 2,553); Global Cognition (n = 2,491).

[c]Model 3 further included adjusted for smoking status, alcohol consumption, work activity, recreational activities, BMI, Mediterranean Diet score, self-reported physician diagnosis of prediabetes or diabetes, self-reported physician diagnosis of coronary heart disease, self-reported physician diagnosis of high blood pressure, and self-reported physician diagnosis of stroke. Sample sizes for each cognitive test were lower because of missing covariates: Consortium to Establish a Registry for Alzheimer's Disease–Immediate Learning (n = 2,532); Consortium to Establish a Registry for Alzheimer's Disease–Delayed Recall (n = 2,531); Animal Fluency Test (n = 2,521); Digit Symbol Substitution Test (n = 2,477); Global Cognition (n = 2,417).
